# Anti-IGF-1R monoclonal antibody inhibits the carcinogenicity activity of acquired trastuzumab-resistant SKOV3

**DOI:** 10.1186/s13048-014-0103-5

**Published:** 2014-11-26

**Authors:** Wei Wang, Yan Zhang, Ming Lv, Jiannan Feng, Hui Peng, Jing Geng, Zhou Lin, Tingting Zhou, Xinying Li, Beifen Shen, Yuanfang Ma, Chunxia Qiao

**Affiliations:** Laboratory of Cellular and Molecular Immunology, Institute of Immunology, Henan University, Kaifeng, 475001 China; Laboratory of Immunology, Institute of Basic Medical Sciences, PO Box 130(3), Taiping Road #27, Beijing, 100850 China; Department of Gynecology and Obstetrics, PLA General Hospital, Fuxing Road No. 28, Beijing, 100853 China; Department of Environment and Pharmacy, Tianjin Institute of Health and Environmental Medicine, Beijing, 100850 China

**Keywords:** IGF-1R, Monoclonal antibody, Acquired resistant, Trastuzumab, Ovarian cancer

## Abstract

**Background:**

Antibody resistance, not only *de novo* but also acquired cases, usually exists and is related with lower survival rate and high risk of recurrence. Reversing the resistance often results in better clinical therapeutic effect. Previously, we established a trastuzumab-resistant ovarian cancer cell line, named as SKOV3-T, with lower HER2 and induced higher IGF-1R expression level to keep cell survival.

**Methods:**

IGF-1R was identified important for SKOV3-T growth. Then, a novel anti-IGF-1R monoclonal antibody, named as LMAb1, was used to inhibit SKOV3-T in cell growth/proliferation, migration, clone formation and *in vivo* carcinogenicity.

**Results:**

In both *in vitro* and *in vivo* assays, LMAb1 showed effective anti-tumor function, especially when being used in combination with trastuzumab, which was beneficial to longer survival time of mice as well as smaller tumor. It was also confirmed preliminarily that the mechanism of antibody might be to inhibit the activation of IGF-1R and downstream MAPK, AKT pathway transduction.

**Conclusion:**

We achieved satisfactory anti-tumor activity using trastuzumab plus LMAb1 in trastuzumab-resistant ovarian cancer model. In similar cases, not only acquired but also *de novo*, good curative effect might be achieved using combined antibody therapy strategies.

## Introduction

An antibody (Ab), also known as an immunoglobulin (Ig), is a large Y-shape protein produced by plasma cells that is used by the immune system to identify and neutralize foreign object, such as bacteria and virus, which is called “antigen”. Monoclonal antibody (mAb) recognizes a unique part of the antigen specifically, e.g. tumor associated antigens like EGFR or HER-2, and these mAbs have been widely used as standard treatment in clinical trails.

Along with more knowledge of the structure and potential modifications of mAbs plays an increasingly important role in cancer immunotherapy. Over the past few years many biological technologies have been invented to prepare therapeutic antibody drugs, including chimeric, humarized or fully human antibodies, radioimmunotherapeutic agents, antibody-drug conjugates (ADCs), and bi-specific T cell engagers (BiTEs), etc. As early as 1998, the FDA approved the use of Trastuzumab, the first anti-HER2 humanized antibody, for HER2-positive breast cancer. In 2012, the FDA approved another anti-HER2 drug, Pertuzumab, for advanced or metastatic breast cancer with high or low expression level of HER2. There has also been a lot of excitement about the development of antibody-drug conjugates, as these drugs are designed to improve local delivery of highly toxic chemotherapeutics meanwhile simultaneously attempting to minimize systemic toxicity. In 2013, an anti-HER2 ADC drug, trastuzumab emtansine, was approved by the FDA for patients with metastatic HER2-positive breast cancer [1].

HER2, a receptor tyrosine-protein kinase also known as erbB-2/CD340, belongs to the transmembrane epidermal growth factor type II receptor family. It represents the prototype of a stable molecular abnormality endowed with well-characterized functional consequences. It has been found in several of the most common solid tumors, including but not limited to ovarian, breast, colon, non-small cell lung cancer, endometrial, prostate and cervical cancer [2-5]. More importantly, HER2 overexpression has been shown to correlate with a worse prognosis in both node-positive and node-negative breast cancer patients. It also has potential therapeutic and diagnostic value in other types of solid tumor, *e.g.* multiple gynecologic cancers [6].

Trastuzumab (Herceptin®, Genentech, CA, USA) is a humanized monoclonal IgG1 antibody that works both through initiation of ADCC and recruitment of NK cells as well as restrain of downstream effectors [7-9]. It was FDA-approved in 1998 as an adjunct to cyclophosphamide, paclitaxel and/or doxorubicin in the treatment of early-stage HER2 positive breast cancer, and as a single drug for adjuvant treatment of early-stage, HER2 positive, high-risk ER/PR-negative breast cancers following multi-modality anthracycline-based therapy [10]. Trastuzumab has provided a promising therapeutic advantage in not only breast cancer but in other tumor types; moreover, combination therapy with trastuzumab and chemotherapeutics is generally more effective than single agents in HER2 positive breast and gastric cancer.

Pertuzumab (Omnitarg®, Genentech, South San Francisco, CA, USA) is a humanized IgG1 mAb. It is a HER heterodimerization inhibitor that binds domain II of the extracellular HER2. Pertuzumab received the US FDA approval for the treatment of HER2-positive metastatic breast cancer on June 8, 2012. Compared to trastuzumab, pertuzumab inhibits a broader array of downstream signal transduction pathways through inhibition of lateral signal transduction [11-15].

Trastuzumab emtansine (Kydcyla/T-DM1, Genentech/Roche) is a novel antibody-drug conjugate approved in 2013 with trastuzumab for targeted delivery and anti-microtubule agent DM1 for cytotoxicity. In contrast to trastuzumab, T-DM1 not only inhibits the growth of cancer cells by binding to the HER2 receptor, but also kills them by emtansine, for emtansine can enter cells and bind to tubulin [16]. T-DM1 has demonstrated robust clinical activity in pretreated HER2-positive breast cancer patients with a 43.6% objective response rate and median PFS of 9.6 months [17]. The global marketing of T-DM1 may over 3 billion in 2018 predicted by Bloomberg Limited Partnership recently.

Although antibody drugs against cancers have made great clinical achievements, there still exist many cases in which the patients do not respond to the antibody at the very beginning; besides, many patients who received antibody treatment relapsed because of subsequent antibody resistance. For instance, many HER2-positive breast cancers do not respond to trastuzumab treatment (de novo resistance), while many trastuzumab-responsive patients develop resistance after continuous trastuzumab infusion within one year (acquired resistance) [18,19]; meanwhile, although the treatments have improved, the major problem in the hematological multiple myeloma (MM) is the resistance to therapy. Most patients will eventually relapse or become resistance to bivatuzumab, which is a humanized anti-CD44v6 variant monoclonal antibody to inhibit cell adhesion to hyaluronan [20,21]; besides, two anti-epidermal growth factor receptor (EGFR) mAbs, the chimeric IgG1 mAb cetuximab and the human IgG2 mAb panitumumab, have shown relevant clinical effect in chemotherapy-refractory metastatic colorectal cancer (mCRC) [22-25]. Because of common resistance to anti-EGFR mAbs, recent guideline recommendations suggest that anti-EGFR mAbs be given only to patients with KRAS wild-type mCRC [26,27]. However, the overall response rate is still not high, ranging from 17% to 60% [28-37].

Antibody resistance phenomenon exists in so many cases that researchers work hard about it, and a lot of articles have been published. The available methods include combination therapy, that is, the mAb was used plus chemotherapy, or radiation therapy, or other mAbs. In a phase III study of women with HER2-positive breast cancer that treated with trastuzumab, the combination therapy with capecitabine and the multi-tyrosine kinase inhibitor lapatinib, which inhibits both HER2 and EGFR, substantially extended progression-free survival time for 4 months [38]. In a randomized clinical trial, breast cancer patients that progressed after previous trastuzumab therapy were recruited. They were treated with trastuzumab plus capecitabine, which provided significant benefit compared with capecitabine alone [39]; Furthermore, in some cases, antibody resistance was dealt with anti-angiogenic agents, e.g. bevacizumab, an anti-VEGF mAb, which can improved the overall survival rate in metastatic colorectal and lung cancers when combined with chemotherapy [40,41], and progression-free survival in metastatic breast and ovarian cancer [42], etc..

In our previous work, an acquired trastuzumab-resistant cell model of human ovarian cancer, SKOV3-T, was established, and IGF-1R molecule was found by microarray analysis and preliminarily testified to be pivotal in cell proliferation. In this study, we confirmed the key role of IGF-1R in SKOV3-T cells compared to SKOV3 in cell growth/proliferation, *in vitro* clone formation, invasion/migration, cell cycling and *in vivo* carcinogenic effect; then a novel anti-IGF-1R human antibody, LMAb1, was prepared and the activity was confirmed to inhibit the carcinogenesis of trastuzumab-resistant ovarian cancer cells both *in vitro* and *in vivo*.

## Methods

### Regents

Trastuzumab (Herceptin®) was obtained from F. Hoffmann-La Roche Ltd.; Antibodies for western blot against EGFR, p-EGFR (Tyr1068), HER2, p-HER2 (Tyr1248), HER3, p-HER3 (Tyr1289), Akt, p-Akt (Ser473), ERK1/2, p-ERK1/2 (Thr202/Tyr204), Src, p-Src (Tyr416), IGF-1R, p-IGF-1R (Tyr1135/Tyr1136), GAPDH and corresponding secondary antibodies were purchased from Cell Signaling Technology; PE conjugated anti-EGFR and anti-HER3, FITC-conjugated Annexin V antibodies and propidium iodide (PI) were from eBioscience; PE conjugatedanti-HER2 antibody was from BD; Electrophoresis reagents and Hybridization Nitrocellulose Filter membranes were from Bio-Rad; BCA protein assay and enhanced chemiluminescent (ECL) reagents were from Pierce; Cell culture medium Dulbecco’s modified Eagle medium (DMEM) and fetal bovine serum (FBS) were purchased from HyClone; Human IGF-1, NRG1-β1/HRG1-β1 was from R & D; IGF-1R expressing plasmid pCMV6-IGF1R was from OriGene; Lentiviral delivery system was packaged by Gene Pharma (China); MTT [3-(4,5-dimethylthiazol-2-yl)-2,5-diphenyl tetrazolium bromide] and agarose (cell culture level) were purchased from Sigma-Aldrich; Matrigel and Transwell chamber was from Millipore. All other chemicals were obtained from commercial source of analytical grade.

### Cell culture

Human ovarian cancer cell line SKOV3 was from ATCC (American Type Culture Collection, ATCC No. HTB-77). The cells were cultivated in DMEM supplemented with 100 units/ml penicillin, 100 units/ml streptomycin, 10% FBS and 4 mM L-glutamine.

Acquired trastuzumab-resistant ovarian cancer cell line SKOV3-T was developed through continuously culturing SKOV3 cells in the presence of 20 μg/ml trastuzumab. Surviving cells were pooled together and tested for dose response to trastuzumab as described before [43]. SKOV3-T cells are now maintained in the presence of 10 μg/ml trastuzumab.

All the cells were incubated in a humidified incubator (Thermo, America) at 37°C with 5% CO2.

### Proliferation assay

The cells were loaded in 96-well plate with 2 × 10^3^ cells per well with or without various concentration of antibody supplemented with 40 ng/ml of IGF-1 for 72 hours. Human IgG isotype was added as negative control. The medium was removed from each well and 10 μL of CCK-8 solution was added to 100 μL medium in each well for 1–4 hours’ incubation at 37°C. The absorbance was measured at 450 nm on a Tecan Spectrophotometer (Tecan SPECTRAFluor, Tecan, Männedorf, Switzerland).

### Transwell assay

Cells were digested to a suspension with a density of 1 × 10^4^/ml. Cells were seeded into the transwell chamber (Millipore, USA) which membrane was coated by a dilution of Matrigel (50 mg/L). The chamber was placed into a 24 well culture plate, with 500 μl of DMEM medium containing 10% serum added outside of the chamber, and 200 μl cell suspension were added in the chamber. After 3, 6, 9, 24 hours, the cells were stained and placed under the fluorescence microscope for observation.

### Agar clone formation assay

Preparation of agarose hydrogels: Agarose was purchased from Sigma-Aldrich, St. Louis, USA. Hydrogel was prepared by dissolving agarose (0.6%, 1.2% w/t) in aqueous solvent at the temperature of 90°C. Once the temperature of this solution is lowered to room temperature, gelation will occur.

Cells were harvested using 0.25% trypsin. counted, and then loaded in agarose scaffolds. Briefly, cells were suspended in 0.6% agarose with the cell density of 1000 cells/1000 μL. The low agarose (2 × DMEM, 10% FBS, 1.2% agarose) molds were allowed to gelation at 4°C for 20 min, and then transferred to 6well culture plate, then add the up agarose (2 × DMEM, 10% FBS, 0.6% agarose).The plate was placed in incubator containing 5% CO2 at 37°C. As control, cell aggregates were cultured with the same condition as that of experiment group. Culture media were changed every 3 days. Assays were performed at time points of 14 days.

### Flow cytometry

Cells were collected and stained with appropriate PE conjugated antibodies against membrane markers. For each sample, data from approximately 15,000 cells were analyzed using a BD-FACStar™ instrument. Data analysis (the percentage and intensity of stained cells, etc.) was performed on a FACS Calibur flow cytometer using the BD CellQuest™ program.

### Western blot

Cell monolayers were washed with cold PBS before they were lysed in cell lysis buffer (20 mM Tris at pH 7.0, 1% Triton-X 100, 0.5% NP-40, 250 mM NaCl, 3 mM EDTA, 3 mM EGTA, 2 mM DTT and protease inhibitor cocktail). Cell lysate supernatants were collected by 12000 × g centrifugation for 15 minutes at 4C°. Total protein concentrations in these fractions were determined by BCA protein assay. The samples were electrophoretically separated on SDS-PAGE and transferred to NC membrane. The membranes were blocked with 5% non-fat dry milk dissolved in TBST (10 mM Tris HCl, 150 mM NaCl containing 0.05% Tween 20, pH 7.4) for 1 hour before probing overnight at 4°C with the appropriate primary antibody (HER1, HER2, HER3, HER4, IGF-1R). In the second day, after washed with TBST three times, the membranes were incubated with HRP-conjugated secondary antibody for 1 hour at room temperature. Signals were detected by X-ray Film following incubation with ECL.

In antibody treating assay, cells were cultivated in 6-well plate and serum starved overnight. In the next day, renew the culture with/without diluted Lmab1 at the concentration of 0.8, 4, 20 μg/ml or 20 μg/ml of trastuzumab for 4 hours.Then cells were stimulated with 20 ng/mL of IGF-I for 20 min. Then cells were collected for western blot analysis.

### Cell cycle

Cells were collected by digestion and prepared by fixation in 75% ethanol overnight (at least 18 hours) at −20°C. Then cells were stained with 50 μg/ml PI and 100 μg/ml RNase A for 30 min at 37°C in the dark. Quantification of the cell cycle distribution was done by flow cytometry analysis.

### Lentivirus systems for down-regulation of IGF-1R

The Lentivirus system was used to knock down IGvirusF-1R expression. The RNAi delivery system was used to deliver shRNAs against IGF-1R as described previously [44].

SKOV3 cells were seeded in a 6-well plate (approximately 5 × 10^4^ cells per well with 2 ml of growth medium). Growth medium was replaced with 2 ml of lentiviral medium containing 8 μg/ml of polybrene at final. After 24 hours, medium was replaced with puromycin-containing growth medium to select transduced cells.

### In vivo carcinogenic and immunotherapy assays

Groups of 5-wk-old female BALB/c athymic, nu/nu (nude) mice were inoculated on fat pad with 2 × 10^6^/0.1 ml SKOV3 or 1 × 10^6^/0.1 ml SKOV3-T cells on day 0. Since day 7, mice bearing palpable tumors were randomized into four groups with 6 mice per group (n = 6). Then mice were observed twice a week about body weight, survival rates and tumor volumes according to the following equation: Tumor volume (mm^3^) =1/2 × (length) × (width)^2^. Pairwise differences between groups were compared.

In *in vivo* immunetherapy assay, mice were inoculated with 1 × 10^6^/0.1 ml SKOV3-T cells. On day 7, mice were treated *i.v.* once a week for four times with 5 mg/kg trastuzumab (group 2), 5 mg/kg Lmab1 (group 3), 2.5 mg/kg Lmab1 (group 4), 5 mg/kg trastuzumab plus 5 mg/kg Lmab1 (group 5), 5 mg/kg trastuzumab and 2.5 mg/kg Lmab1 (group 6), natural saline (N.S.) were set as negative control (group 1).

Care, in animal assays, we followed the Guidelines for the welfare and use of animals in cancer research. Use and treatment of mice were in strict agreement with international guidelines for the care and use of laboratory animals and approved by Animal Ethics Committee of Institute of Basic Medical Sciences.

## Results

### Acquired trastuzumab-resistant SKOV3-T cells grow faster than SKVO3

**Figure 1:**
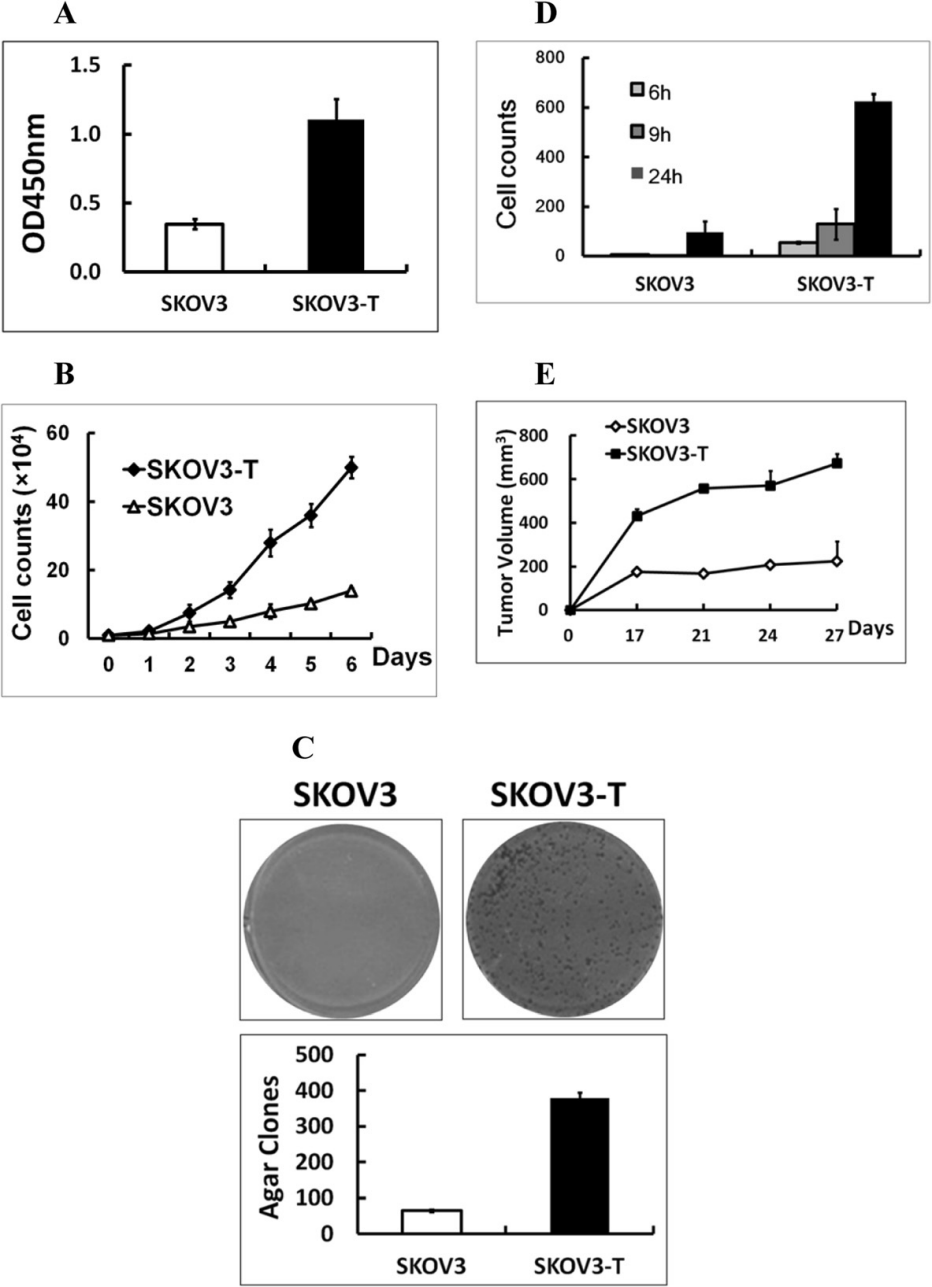
**Acquired trastuzumab-resistant cell line SKOV3-T cells grow faster than SKOV3.** SKOV3 cells were cultivated for 8 months in the presence of 20 μg/ml trastuzumab continuously to obtain SKOV3-T cells. The comparison of SKOV3-T and parental SKOV3 cells by **(A)** cell proliferation, **(B)** cell counting, **(C)** agar clone formation, **(D)** transwell, and **(E)** *in vivo* carcinogenic assays. In cell counting assays, cells were cultured in day 0 at the start concentration of 1 × 10^4^ per 24-well, and in day 1 to day 6, the cells were digested everyday and the whole cell number was counted. The trastuzumab-resistant SKOV3-T seemed to have significantly enhanced cell growth/proliferation, clone formation, stronger invasion and migration character both *in vitro* and *in vivo* versus non-resistant SKOV3 cells.

Human ovarian cancer SKOV3 cells, which over express HER2, werecultured continuously for 8 months in the presence of 10 μg/ml trastuzumab, resulting in the acquisition of trastuzumab resistance in the surviving cell population. Compared with the parental cells, the resistant SKOV3 cells had a significant higher viability or proliferative capacity in cell proliferation assay (Figure 1A) in 96-well plate and cell counting assay (Figure 1B); meanwhile, SKOV3-Tdisplayeddramatically increased colony formation on the agar cloning assay, for the clones were obviously bigger and much more than SKOV3 (Figure 1C); furthermore, in transwell assays, SKOV3-T exhibited stronger migration capacity, for after 24 hours, about 626 SKOV3-T cells moved while only less than 100 SKOV3 cells moved across the well (Figure 1D); furthermore, in *in vivo* carcinogenic assay, the mean volume of SKOV3-T transplanted tumor was significantly larger than SKOV3 (Figure 1E). These results suggest that trastuzumab-resistant ovarian cancer cells SKOV3-T growth much faster and migrate better than SKOV3, suggesting stronger malignancy and metastasis character of SKOV3-T*in vivo* than non-resistant cells.

### IGF-1R can promote the proliferation of SKOV3

**Figure 2:**
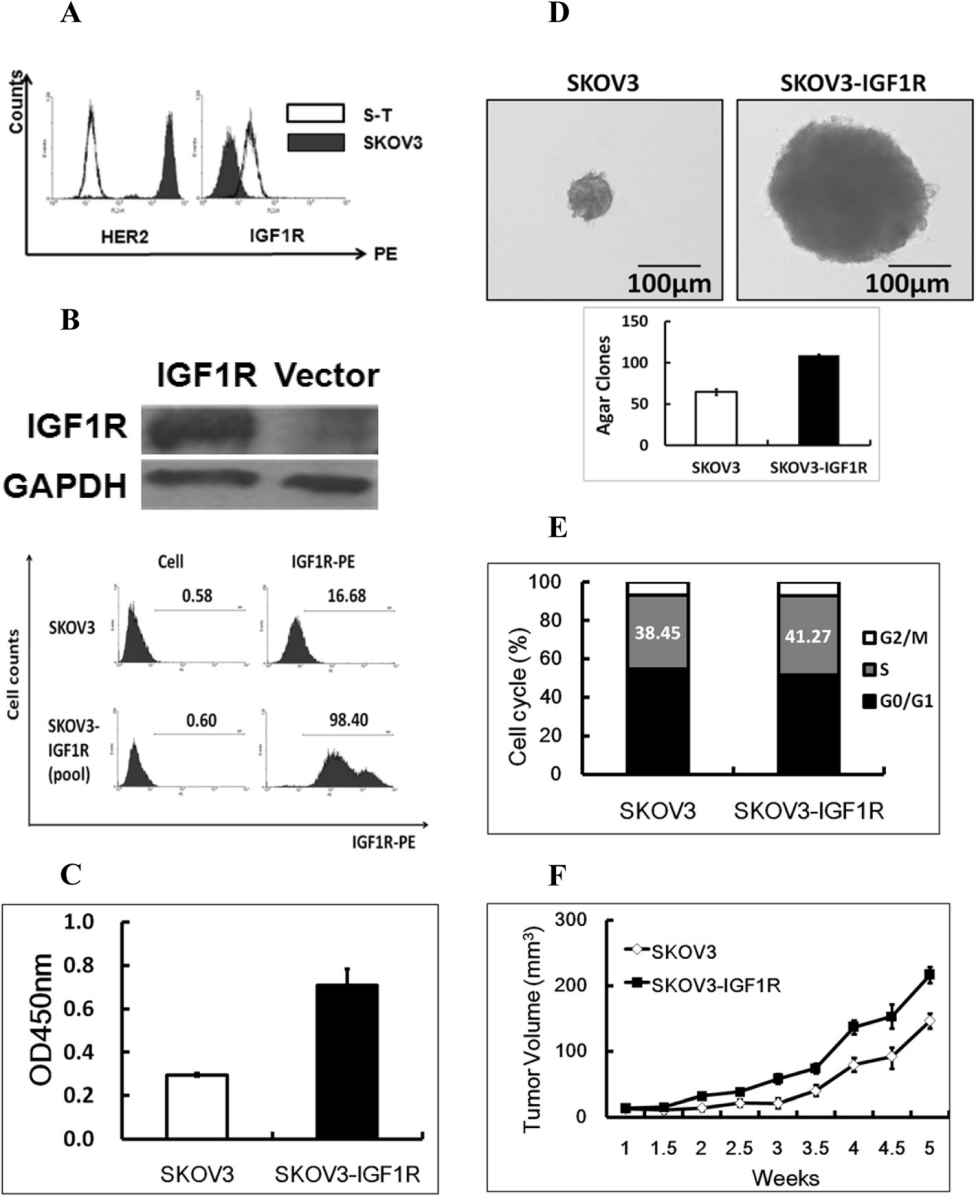
**IGF-1R can promote the proliferation of SKOV3. (A)** IGF-1R expression was up-regulated in SKOV3-T while HER2 was opposite by flow cytometry analysis; **(B)** IGF-1R-positive SKOV3 cell preparation by eukaryotic transfection with pCMV6-IGF-1R plasmid. Cell proliferation **(C)** and agar clone formation **(D)** assays both indicated the enhanced carcinogenic activity of IGF-1R-positive SKOV3 cells, while according to cell cycle analysis **(E)**, SKOV3-IGF1R owned more S-phase cells in order to multiply quicker; Similarly, *in vivo* tumor model **(F)** further displayed more rapid tumor growth of SKOV3-IGF1R, indicating that IGF-1R can promote the cell survival and multiplication in ovarian cancer SKOV3 cells. To be clear, in this figure, “SKOV3” sample means original pCMV6 transfected cells.

According to our previous work, based on the mRNA array analysis, IGF-1R up-regulation (Figure 2A) was proved to play a key role in SKOV3-T proliferation *in vitro*. To the opposite, the expression level of HER2 in SKOV3-T was dramatically lower than SKOV3by flow cytometry method (Figure 2A).To further examine the role of IGF-1R in ovarian cancer cells, IGF-1R-pCMV6 plasmid, a eukaryotic expression vector subcloned with full IGF-1R exon sequence, was transfected into SKOV3 using lipofectamine 2000, generating a pool of IGF-1R-positive SKOV3 cells (Figure 2B). As shown in Figure 2C, the transfected cells had much higher viability or proliferative capacity than SKOV3; meanwhile, they also displayed increased colony formation capacity (Figure 2D); furthermore, according to the cell cycling assay, SKOV3-IGF-1R cells (pool) has S-phase cells than SKOV3, suggesting much quicker cell multiplication rate of SKOV3-IGF-1Rcells (Figure 2E); in *in vivo* tumor formation assay, IGF-1R positive cells exhibited more rapid growth capacity than parental cells (Figure 2F), indicating thatIGF-1R can promote the proliferation of SKOV3. All above demonstrated the importance of membrane IGF-1R as well as its downstream cascade in retaining/promoting the survival of SKOV3-T, especially when HER2-related signal pathway was down-regulated.

### IGF-1R knockdown by shRNA could inhibit the proliferation of SKOV3-T

**Figure 3:**
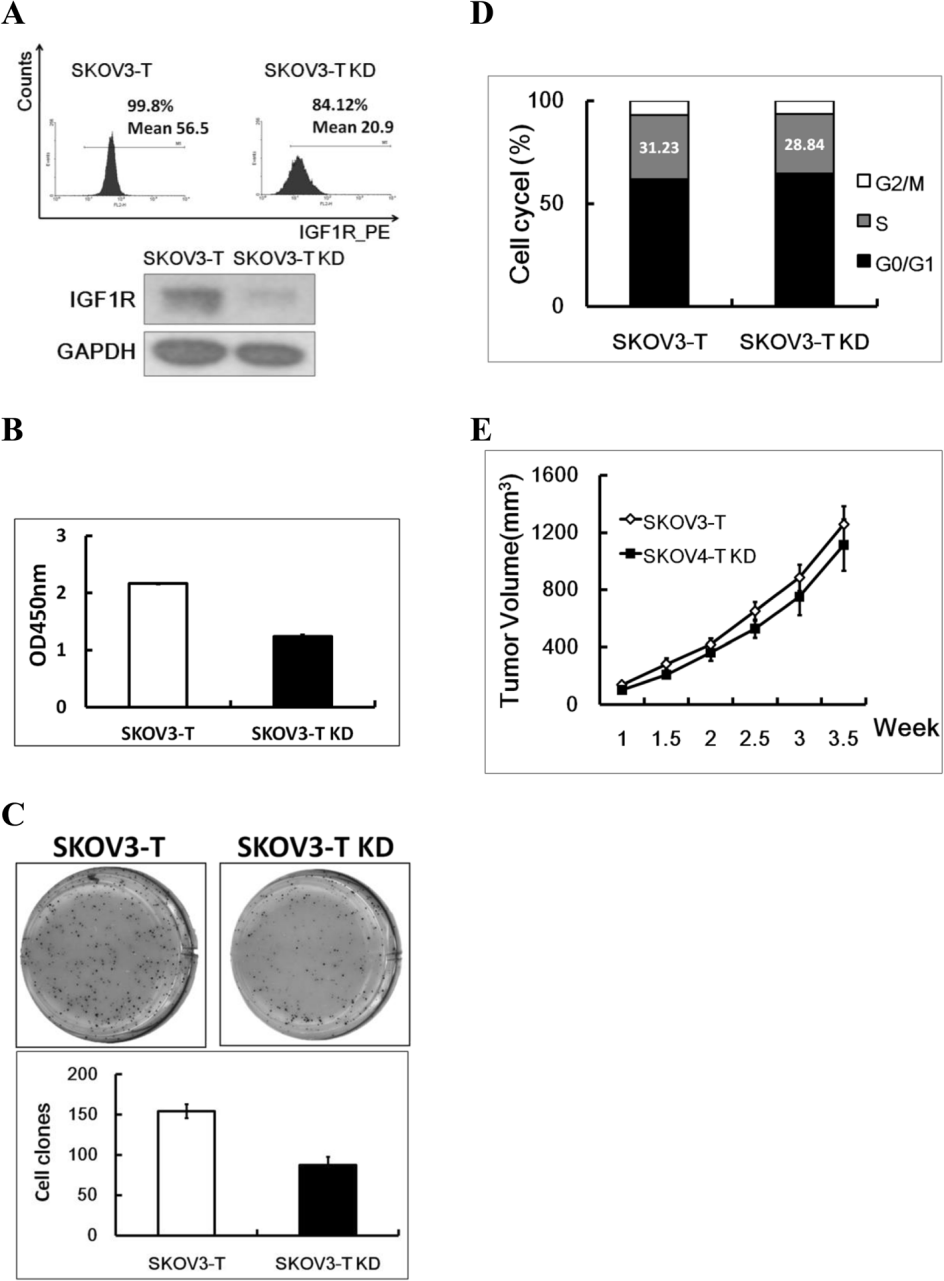
**IGF-1R knockdown could inhibit the proliferation of SKOV3-T. (A)** IGF-1R expression was knocked down in SKOV3-T cells using lentivirus system and cells were analyzed by flow cytometry (up panel) and western blot (down panel) analysis; Cell proliferation **(B)** and clone formation **(C)** analysis both indicated that in SKOV3 KD cells, the cell growth was inhibited, while according to cell cycle analysis **(D)**, SKOV3-T KD cells owned less S-phase cells in order to slower cell multiplification; In *in vivo* carcinogenic model, SKOV3-T KD exhibited slower tumor growth rate contrasting to SKOV3-T **(E)**, indicating that IGF-1R was important to SKVO3-T cells when HER2-related cascade was blocked. To be clear, in this figure, “SKOV3-T” sample means control virus treated SKOV3-T cells.

As shown above, IGF-1R could fasten the cell growth of SKOV3, which was similar to the quick growth of SKOV3-T. In order to further analyze the biofunction(s) of IGF-1R in SKOV3-T cells, a lentivirus vector to knock down IGF-1R was packed and transduced into SKOV3-T cells, while cells transduced with virus CON054 were set as negative control. As shown in Figure 3A, IGF-1R expression was inhibited according to flow cytometry and western blot analysis. Furtherly, cell proliferation assay showed that SKOV3-T KD cells grow more slowly than SKOV3-T (Figure 3B); similarly, the agar clone formation capacity of SKOV3-T KD cells was weaker (Figure 3C); meanwhile, in the cell cycling assay shown in Figure 3D, SKOV3-T KD exhibited less S-phase cells (28.84%) than SKOV3-T (31.23%), indicating thatIGF-1R could affect the cell cycle, thus influence the cell proliferation of SKOV3-T. Further *in vivo* experiment also displayed the importance of IGF-1R in SKOV3-T, for the mean tumor volume of SKOV3-T was ~1257 mm^3^, while SKOV3 KD was ~1115 mm^3^ (Figure 3E).

### Anti-IGF-1R mAb (LMAb1) could inhibit the proliferation of SKOV3-T

**Figure 4:**
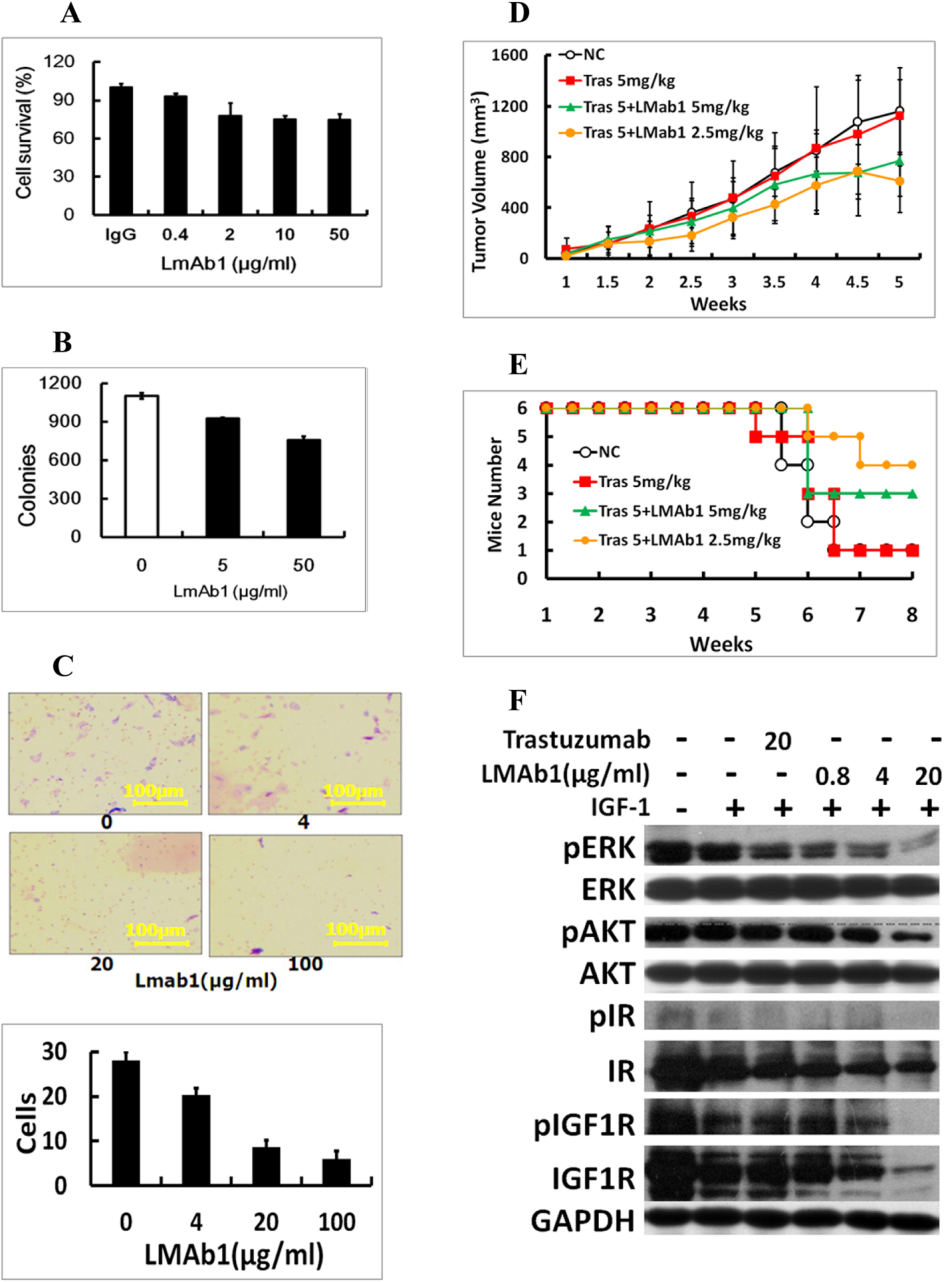
**LMAb1could inhibit the proliferation of SKOV3-T. (A)** LMAb1 could inhibit cell proliferation of SKOV3-T at a dose dependent manner. Similarly, the character of clone formation **(B)** and invasion/migration identified by transwell assay **(C)** could also be inhibited by LMAb1 in resistant SKOV3-T cells; **(D & E)** *in vivo* immunotherapy of LMAb1 combined with/without trastuzumab to SKOV3-T xenograft model in nude mice. **D**: mean tumor volume and **E**: overall survival rate; **(F)** LMAb1 could inhibit IGF-1R signal pathway transduction, for it could inhibit the MAPK and AKT activation stimulated by IGF-1. Presumably, for IGF-1R was dramatically up-regulated in acquired trastuzumab-resistant SKOV3-T cells, specific anti-IGF-1R antibody (LMAb1) could block the IGF-1R-driven signal cascade in order to help slower cell growth, reduce clone formation, shorten S-phase, and inhibit invasion and migration of cells.

Since the trastuzumab resistant SKOV3-T cells have higher IGF-1R level than parental SKOV3 cells, a novel anti-IGF-1R mAb, named as LMAb1, was screened out from a natural fully human phage library in our lab (Chinese patent: 201410271608.8). Here, in SKOV3-T cells, LMAb1 could inhibit cell proliferation, for in 10 μg/ml LMAb1 treated samples, cell survival rate was ~75% contrasting to non-treated groups (Figure 4A); meanwhile, the agar clone formation of SKOV3-T was also inhibited by LMAb1. When the concentration of antibody reached 50 μg/ml, the average clone number was ~757 contrasting to ~1102 of SKOV3-T (Figure 4B); furthermore, in transwell assay, the migration capacity was inhibited on a dose dependent manner, for after 15 hours, about 300 SKOV3-T cells per well were migrated, while less cells moved across the hole in antibody treated samples. Here, four different scales of each sample were photographed and counted. Contrasting to the control sample (the mean cell number of each scale was about 30), the 100 μg/ml antibody treated sample showed less than 10 migrated cells (Figure 4C); In *in vivo* experiments, LMAb1 showed certain anti-tumor capacity, especially in the groups treated with LMAb1 combined with trastuzumab. In contrast to the SKOV3-T group with the mean tumor volume of ~1161 mm^3^ and the trastuzumab treated group (~1123 mm^3^), the groups administrated with LMAb1, whether alone or plus trastuzumab, has the mean tumor volume of 600 ~ 700 mm^3^ (Figure 4D). More interestingly, the mean survival time of LMAb1 plus trastuzumab treated mice was much longer. As shown in Figure 4E, in week 8, about half of LMAb1 plus trastuzumab treated mice survived, while there was only one or fewer mice was still alive in other groups. For the IGF-1R is essentially expressed by most organs and tissues, therefore anti-IGF-1R antibody such as LMAb1 might have side-effects, which may influence the survival rate of mice as well as the anti-tumor effects. According to the cell signaling assays shown in Figure 4F, LMAb1 could block the IGF-1 induced activation of pERK, pAKT and pIGF-1R along with the increase concentration of LMAb1, indicating that inhibition of PI3K-AKT as well as MAPK cascade might be one of the anti-tumor mechanisms of anti-IGF-1R antibody LMAb1.

## Discussion

There are more and more antibody resistance clinical cases, while many patients with cancer do not respond to antibody treatment (*de novo* resistance). More and more patients who receive antibody treatment have the problem of the relapse and antibody resistance (acquired resistance). Some acquired antibody resistant cases showed low recurrence-free survival, cancer-related survival, and/or overall survival (OS), and high risk of local and distant recurrence.

Type I insulin-like growth factor receptor (IGF-1R) has been founded for decades for its role in growth and tumorigenesis [45]. Not until recently had advances in medicinal chemistry and biotechnology provided the tools for targeting the insulin-like growth factor (IGF) pathway in patients. IGF-1R belongs to the insulin receptor (IR) family that includes the IR, IGF-1R, IGF-1R/IR, and the mannose 6-phosphate receptor (also known as IGF-2R). IGF-1R can be activated by the ligands insulin-like growth factor-1 (IGF-1) or insulin-like growth factor-2 (IGF-2). Intracellular signaling of IGF-1R is mediated through IR substrates and Src-homology collagen protein (Shc) [46], which leads to activation of the mitogen-activated protein kinase (MAPK) pathway and the PI3K-AKT pathway [47]. IGF-1R is ubiquitously expressed in normal tissues and plays an important role in growth and various physiological functions, including those involving the cardiac and neurological systems, as well as glucose homeostasis. The influence on glucose probably occurs through feedback down-regulation of HGH by circulating IGF-1 and the local effect of IGF-1 on IGF-1R in the muscles or kidneys to promote glucose uptake [48,49].

Extensive *in vitro* and *in vivo* studies have implicated IGF-1R, IGF-1, and IGF-2 signaling in cancer development, progression, and maintenance. IGF-1R expression is critical for anchorage-independent growth, a well identified property of cancer cells. IGF-1 and IGF-2 are strong mitogens in a variety of cancer cell lines, including breast cancer [50-53], colon cancer [54,55], prostate cancer [56], and myeloma [57]. High circulating levels of IGF-1 have been associated with increased risk of prostate, breast, and colon cancers [45]. The IGF/IGF-1R pathway have been shown to have extensive cross-talk with the epidermal growth factor receptor (EGFR), estrogen receptor (ER) and human epidermal growth factor receptor 2 (HER2) signaling pathways and to play an important role in the resistance mechanisms of EGFR/HER2-targeted agents and cytotoxic drugs [58]. Recent work also indicated a potential role for IGF-1R in the resistance to RAF-MEK inhibitors [59] and mTOR inhibitors [60], however, there were rare report to show the importance of IGF-1R in antibody-resistant cases. IGF-1R can be founded in most solid tumors and hematological malignancies examined to date, and IGF-2 overexpression, IGFBP modulations, and IGF-2R down-regulation have also been founded in cancer cells [46,61,62]. Nevertheless, unlike other growth factor receptors such as EGFR and HER-2, activating mutations of the IGF-1R gene have not been reported, and gene amplification is rare in the tumors that have been tested [63]. On the other hand, several genetic abnormalities can lead indirectly to IGF/IGF-1R overexpression and signaling. Some tumors, including hepatocellular carcinoma and breast cancer, have been associated with loss of heterozygosity of the *IGF2R* gene [64]. Loss of imprinting of IGF-2, first described in Wilms tumor, has since been identified in adult tumors and is associated with an increased risk of colon cancer [65,66]. These genetic changes may increase IGF-2 production or its bioavailability for IGF-1R signaling.

At least seven human or humanized anti–IGF-1R mAbs entered clinical trials: Cixutumumab [67-69], Figitumumab [70-74], Dalotuzumab [75-77], Ganitumab [78-81], R1507 [82], SCH. 717454 [83], AVE1642 [84,85] and BIIB022 [86], etc.. Common mechanisms of antibody action include blockade of the receptor from ligand binding and internalization/degradation of IGF-1R [87]. In addition, anti-IGF1R mAbs also down-regulate the IGF-1R/IR hybrid receptor [88]. Common treatment have emerged adverse events that include hyperglycemia.

IGF-1R signaling has been causally linked to de novo or acquired resistance to trastuzumab and EGFR-targeting agents in a lot of models. *In vitro* and *in vivo* tumor models have also demonstrated direct interactions between IGF-1R, EGFR/HER-2 [61,85-88], and co-localization of IGF-1R and HER-2. Treatment of resistant cells with IGF-1R inhibitors was shown to inhibit transactivation of HER2 and restore sensitivity to trastuzumab [89].

Our previous study reported the trastuzumab-resistant ovarian cancer cells, SKOV3-T, with lower HER2 and higher IGF-1R and HER3 expression level than parent SKOV3 cells. The two new biomarkers were suggested to be important in maintaining the cell growth [43]. According to our work, in SKOV3-T cells, epitope escaping might exist during long-term treatment with trastuzumab, which was possibly the main reason why SKOV3-T possessed resistant capacity to trastuzumab. When full length of HER2 gene was transfected into SKOV3-T, the sensitivity to trastuzumab could be recovered (data not shown); meanwhile, the IGF-1R expression level was up-regulated in SKOV3-T cells, which should be important to keep cell survival at the absence of HER2 (Figure 1). Here, we identified the role of IGF-1R in cell growth/proliferation, migration, cell cycling, clone formation, and *in vivo* carcinogenic character (Figures 2 and 3); Based on the data above, we prepared an IGF-1R mAb called LMAb1 to treat SKOV3-T. LMAb1 showed curative effect against the resistant cells; *In vivo* assays showed its effective anti-tumor function, especially when being used in combination with trastuzumab, which was beneficial to higher survival rate of mice as well as smaller tumor. It was inferred that trastuzumab might ease the side-effect of LMAb1 *in vivo* with unknown reason, for mice treated with LMAb1 only didn’t contribute to obvious longer survival time, although the tumor volume was indeed smaller than control group(s); besides, we also evidenced preliminarily that the mechanism of antibody included the inhibition of IGF-1R and downstream MAPK, AKT pathway activation (Figure 4). Similarly, we also use LMAb1 to treat MCF-7 cells, an IGF-1R-positive breast cancer cell line, which showed satisfactory anti-tumor activity by flow cytometry, cell proliferation and transwell *in vitro* (data not show).

## Conclusion

In conclusion, we achieved satisfactory anti-tumor activity with combination therapy strategy, e.g. trastuzumab plus anti-IGF-1R mAb (LMAb1), in trastuzumab-resistant ovarian cancer model. According to our work, it should be inferred that in similar cases with resistance to a single antibody drug, not only acquired but also *de novo*, combination therapeutic strategies might achieve better curative effect.
